# Effects of Developmental Exposure to 2,2′,4,4′,5-Pentabromodiphenyl Ether (PBDE-99) on Sex Steroids, Sexual Development, and Sexually Dimorphic Behavior in Rats

**DOI:** 10.1289/ehp.8391

**Published:** 2005-10-06

**Authors:** Hellmuth Lilienthal, Alfons Hack, Astrid Roth-Härer, Simone Wichert Grande, Chris E. Talsness

**Affiliations:** 1 Department of Neurobehavioral Toxicology, Medical Institute of Environmental Hygiene, Heinrich Heine University, Düsseldorf, Germany; 2 Center of Clinical Research, Ruhr University, Bochum, Germany; 3 Institute of Clinical Pharmacology and Toxicology, Department of Toxicology, Charité University Medical School, Berlin, Germany

**Keywords:** ovarian follicles, polybrominated diphenyl ether, polychlorinated biphenyls, rats, sex steroids, sexual development, sexually dimorphic behavior

## Abstract

Increasing concentrations of polybrominated flame retardants, including polybrominated diphenyl ethers (PBDEs), in breast milk cause concern about possible developmental effects in nursed babies. Because previous studies in rats have indicated effects on sex steroids and sexually dimorphic behavior after maternal exposure to polychlorinated biphenyls (PCBs), our goal in the present study was to determine if developmental exposure to 2,2′,4,4′,5-pentabromodiphenyl ether (PBDE-99) induces similar endocrine-mediated effects. Pregnant rats were exposed to vehicle or PBDE-99 (1 or 10 mg/kg body weight, daily during gestational days 10–18). For comparison, we also included a group exposed to the technical PCB mixture Aroclor 1254 (30 mg/kg body weight, daily). PBDE exposure resulted in pronounced decreases in circulating sex steroids in male offspring at weaning and in adulthood. Female offspring were less affected. Anogenital distance was reduced in male offspring. Puberty onset was delayed in female offspring at the higher dose level, whereas a slight acceleration was detected in low-dose males. The number of primordial/primary ovarian follicles was reduced in females at the lower dose, whereas decline of secondary follicles was more pronounced at the higher dose. Sweet preference was dose-dependently increased in PBDE-exposed adult males, indicating a feminization of this sexually dimorphic behavior. Aroclor 1254 did not alter sweet preference and numbers of primordial/primary and secondary follicles but it did affect steroid concentrations in males and sexual development in both sexes. PBDE concentrations in tissues of dams and offspring were highest on gestational day 19. These results support the hypothesis that PBDEs are endocrine-active compounds and interfere with sexual development and sexually dimorphic behavior.

Several groups of polyhalogenated aromatic hydrocarbons, including dioxins and poly-chlorinated biphenyls (PCBs), are known to induce developmental toxicity (e.g., Peterson et al. 1993; Schantz 1996). These compounds also exert endocrine-modulating effects that are likely to underlie some of their developmental actions (e.g., Gore 2001; Li et al. 1997; Porterfield 2000; Schantz and Widholm 2001). For instance, a mixture of PCBs reconstituted according to the congener pattern found in human breast milk has been shown to cause reductions in circulating sex steroids and alterations in sexual differentiation of the brain and sexually dimorphic behavior in rats (Hany et al. 1999; Kaya et al. 2002). Although concentrations of dioxins and PCBs in environmental samples are decreasing, marked increases have been reported for brominated flame retardants in recent years and, in particular, for polybrominated diphenyl ethers (PBDEs) (Noren and Meironyte 2000; Schecter et al. 2003; Sjödin et al. 2004). The PBDE molecule has two phenol rings linked by an oxygen atom and a variable number of bromine substituents that can bind at 10 different positions. Depending on the number and position of bromine atoms, 209 different congeners can be formed, but only a few of them are present in commercial mixtures. For instance, the major constituents of the commercial PBDE mixture DE-71 are pentabrominated PBDE-99 (2,2′,4,4′,5-pentabromodiphenyl ether) and tetrabrominated PBDE-47, which together account for approximately 85% of the mixture. The remaining 15% of the mixture consists mainly of pentabrominated PBDE-100 and, in decreasing proportions, the hexabrominated congeners PBDE-153 and PBDE-154 (Sjödin et al. 2003).

Knowledge about developmental toxicity of PBDE is limited (Birnbaum and Staskal 2004), but initial results indicate that DE-71 causes effects on thyroid hormones, sex steroids, and development of reproductive organs (Stoker et al. 2004; Zhou et al. 2002). Exposure to single PBDE congeners in mice affects neurobehavioral development (Branchi et al. 2002, 2004; Viberg et al. 2004) and cholinergic receptors in the brain (Viberg et al. 2002, 2003) in a similar fashion to that described after developmental exposure to PCBs in rats and mice (Brouwer et al. 1995; Eriksson and Frederiksson 1998). The purpose of the present study was to evaluate whether PBDE-99, which is one of the most abundant PBDEs in the environment, also affects end points that have been shown to be changed by the reconstituted PCB mixture, namely, circulating steroids, steroid-dependent behavior, and reproductive organ development. We chose sweet preference to examine sexually dimorphic behavior because a marked sex difference is observed in adult rats, with males consuming smaller amounts of sweetened solutions than females (Valenstein et al. 1967). This behavior has been shown to be sensitive to perinatal exposure to nicotine (Lichtensteiger and Schlumpf 1993) or a reconstituted PCB mixture (Hany et al. 1999; Kaya et al. 2002). Both treatments caused elevated sweet preference in adult males, along with a reduction of aromatase activity observed in the brain of newborn male pups (Hany et al. 1999; Lichtensteiger and Schlumpf 1993). Precise measurement of sweet preference is easy to conduct and can be determined in home cages without handling the animals. We also evaluated ovarian follicle numbers in adult female offspring because alterations in folliculogenesis have been observed after exposure to the commercial PCB mixture Aroclor 1016 (Baldridge et al. 2003). Changes in follicular maturation provide an indication of direct effects on the ovary or indirect effects due to disturbance of the hypothalamic-pituitary-ovary axis that may occur after exposure to PBDE-99. For comparison, we included a group exposed to the commercial mixture Aroclor 1254 (A1254) in the present study.

## Materials and Methods

### Animals and treatments.

All procedures involving rats were approved by the local authorities according to the German legal requirements for animal experiments (Tierschutzgesetz, Bundesgesetzblatt 1998, I.S. 1105). Animals used in the study were treated humanely and with regard for alleviation of discomfort and suffering. We obtained pregnant Long-Evans hooded rats (*n* = 108) from the breeder (Janvier, La Geneste St. Isle, France). They were housed in single plastic cages with a bedding of sawdust. The animals were exposed to 12-hr light/dark periods starting with lights on at 0600 hr. The ambient room temperature was 23°C, and the relative humidity was 55%. Food and water were available *ad libitum*. Weight-matched groups of dams (*n* = 27/group) received daily subcutaneous injections of vehicle (olive oil), PBDE-99 at doses of 1 or 10 mg/kg body weight (BDE01 and BDE10 groups, respectively), or the technical PCB mixture Aroclor 1254 (A1254; lot W-135-11) at a dose of 30 mg/kg body weight from gestational day (GD) 10 through GD18. The doses of PBDE-99 used in this study were based on a previous report describing neurobehavioral effects of this congener in mice (Eriksson et al. 2001). The doses selected take into account the longer treatment period and the maternal exposure in our rats, in contrast to the single exposure and direct treatment of neonatal mice. The dose of A1254 is in the same range as the dose previously used in our laboratory for the comparison with the reconstituted PCB mixture (Hany et al. 1999), when adjusted for the shorter exposure period in the present study. Chemicals were obtained from Promochem (Wesel, Germany). The PBDE-99 (lot 001) had a purity of approximately 99% and was analyzed with high-resolution gas chromatography (GC)/high-resolution mass spectrometry (MS) by a commercial analytical laboratory (Oekometric, Bayreuth, Germany) for polybrominated dioxins, polybrominated dibenzofurans, and coplanar polybrominated biphenyls. All contaminants were below the detection limits: 0.05 mg 2,3,7,8-tetrabromodibenzo-*p*-dioxin/kg PBDE-99 and 5 mg octabromodibenzo-*p*-dioxin/kg PBDE-99. For the respective chlorinated compounds, this would yield a maximum total toxic equivalent (TEQ) of 0.50 mg/kg PBDE-99, calculated as North Atlantic Treaty Organization TEQ for dibenzo-*p*-dioxins and dibenzofurans, and of 0.01 mg/kg PBDE-99, calculated as World Health Organization TEQ for biphenyls.

On GD19, three dams per group were sacrificed, and blood was taken by heart puncture for hormone analysis. Blood samples were centrifuged twice at 3,500 rpm for 15 min, and the supernatant stored at –30°C until analysis. Maternal pituitaries and brains were collected and weighed. Brains and perirenal adipose tissue were deep-frozen until determination of maternal PBDE concentrations. The fetuses were counted and sacrificed, and their brains were removed and pooled on a litter basis for determination of PBDE concentrations in offspring. On the day of birth, which was assigned postnatal day (PND) 0, litter sizes and weights were determined and the pups were examined for gross malformations. There was no general standardization of litters; however, litters with > 10 pups were culled to 10, and litters with < 5 pups were not used. Measurement of litter weights was repeated on PNDs 7, 14, and 21. At weaning (PND21), necropsies were repeated in randomly selected dams and offspring. Blood was taken from eight dams per group and one male and one female pup from each of their litters for hormone analyses. Brains and perirenal adipose tissue from dams and offspring were deep-frozen for PBDE analyses. Uteri of the dams were examined for the number of implantations. The pituitary, thymus, testes, ventral prostate, and uterus were removed from pups to determine organ weights. After weaning, the offspring were separated by sex and housed in group cages (four or five per cage) until the beginning of the behavioral tests. Dissections were repeated in adult offspring and included the removal of thyroid glands, and removal of ovaries to determine follicle numbers. Dams and offspring were randomly assigned to all treatments, dissections, and measurements. The only exception was that dams were weight-matched before they were assigned to one of the exposure groups to prevent possible confounding of PBDE-99 effects by differences not related to exposure.

### Sexual development.

We measured anogenital distance, a marker of sexual development, in weanling pups and adult offspring used for dissections. We did not measure anogenital distance in neonatal pups because of the limited precision of the smaller absolute values. In addition, the onset of puberty was determined by daily examination of female pups from PND30 onward for vaginal opening and of male pups for balanopreputial separation starting on PND40. For this, one to five pups of either sex were used per litter, depending on the number of available male and female pups.

### Analysis of sex steroids.

We used commercial enzyme-linked immunosorbent assay (ELISA) kits (DRG, Marburg, Germany) for determination of serum concentrations of 17β-estradiol and testosterone. Because these kits were developed for human serum, we used a previously established method (Hany et al. 1999) that involved extraction and enrichment of steroids from rat serum in diethyl ether to avoid protein matrix effects and to obtain an appropriate concentration range. To measure for specificity, we determined recovery rates in pooled sera (98–105% for testosterone and 105–112% for 17β-estradiol). Recovery rates > 100% were due to residual cross-reactivity with structurally similar steroids. In the case of estradiol, the zero standard of the ELISA kit contained some residual 17β-estradiol that was revealed during the extraction and enrichment procedure.

### Ovarian follicles.

Whole ovaries (*n* = 8 for each group) were fixed in Bouin’s solution, dehydrated in ethanol, and embedded in paraffin. Serial sections were made every 6 μm and stained with hemotoxylin and eosin. Primordial and primary follicles were counted in five sections, 240 μm apart, taken from the middle of the ovary. Every tenth section (60 μm apart) of the entire ovary was analyzed to evaluate the numbers of secondary, tertiary, and atretic follicles. To avoid duplication, we counted only follicles in which the nucleolus could be seen.

Ovarian follicles were classified according to a modification (Plowchalk et al. 1993) of a previously established scheme (Pedersen and Peters 1968). Primordial follicles and primary follicles were counted together and were identified by oocytes surrounded by a single layer of either squamous or cuboidal epithelial cells. The oocytes of secondary follicles were surrounded by more than one layer of granulosa cells or by one layer plus an incomplete second layer with signs of theca interna development. Tertiary follicles were distinguished by antral formation. Atretic follicles were characterized by the appearance of pyknotic granulosa cells, disorganized granulosa cells, and detachment from the basement membrane, a degenerating oocyte surrounded by an envelope of degenerating cumulus cells or loss of cumulus cells (Borgeest et al. 2002; Devine et al. 2000).

### Sweet preference test.

The method to determine sweet preference was performed as described previously by Kaya et al. (2002). Starting on PND120, 9–11 males and 10–12 females per group (not more than 1 male and 1 female taken from a litter) were housed in single cages and each was given two bottles filled with tap water. During this adaptation period of 7 days, basal water intake was determined. After adaptation, each rat was given a bottle filled with tap water and another bottle filled with 0.25% saccharin solution in water (Fluka, Seelze, Germany). Position of the bottles was counterbalanced in each group and changed on every second day to prevent position preferences. Solutions were prepared daily, and tap water was also changed each day during the test period of 5 days. Food was available *ad libitum* throughout the testing time. Weight of rats was determined on the day before and on the day after the test. The ratio of saccharin to water intake was used to determine sweet preference. The test was conducted by an investigator who was unaware of the exposure conditions.

### Analysis of PBDE-99 in tissues.

A sub-sample of brain and adipose tissue was homogenized and extracted with a 7:2 mixture of hexane:isopropanol. The mixture was centrifuged, and an aliquot was shaken with concentrated sulfuric acid to remove fatty components. The extract was then analyzed by capillary GC with MS in single-ion detection mode (SIM) to establish approximate concentrations of PBDE-99. These rough estimates were the basis for the further preparation of the samples. An internal standard, either PCB-209 or ^13^C-labeled PBDE-99 was added to the extract. The mixture was shaken, centrifuged, decanted, and concentrated. For the cleanup, this concentrated extract was poured onto a combination of two solid-phase extraction (SPE) columns. The first SPE column (SiOH–H) contained a mixture of silica phase impregnated with sulfuric acid and a strongly acidic cation exchanger based on silica with benzene sulfonic acid modification. This SiOH–H column was used together with a silica-phase (SiOH) column. For analysis, the cleaned extract was diluted or concentrated depending on the estimated PBDE-99 concentration in the tissue. Instrumental analysis was performed using capillary GC with an electron capture detector. If the result was uncertain, analysis was performed again using GC/MS-SIM. In this case we used ^13^C-PBDE as the internal standard. For quality control, two samples consisting of neat bacon fat and bacon fat spiked with native PBDE-99 were analyzed in each series of determinations (10–15 samples). The detection limit was 5 ng PBDE-99/g lipid, and the recovery rate was 100% based on the analysis of the spiked bacon fat.

### Statistical analysis.

We used the SAS statistical package (version 6.2; SAS Institute, Cary, NC, USA) for all statistical evaluations. The litter was the statistical unit in analyses of all data in offspring, and sex was nested within litter. We analyzed the development of body weights in pregnant dams by two-way analysis of variance (ANOVA) with repeated measures and exposure as the between factor and time as the within factor. After significant main effects, we performed post hoc comparisons of groups with the Ryan-Einot-Gabriel-Walsh test. Development of body weights in offspring was analyzed with three-way ANOVA with repeated measures (exposure as between-litter factor and sex and time as within-litter factors). This was followed by preplanned separate analyses in male and female pups. In addition, we analyzed these data with a pre-planned analysis of covariance with litter size as the covariate. The ratio of organ to body weight was calculated to yield relative organ weights, and the resulting values were analyzed by one-way ANOVA with exposure as the between factor. One-way ANOVA was also used for analyses of steroid concentrations with exposure as the between factor. To evaluate data on vaginal opening and preputial separation, we calculated a mean across the values obtained in one litter, so that these statistics were entirely litter based. Results were evaluated with a nonparametric test (van der Waerden test using normalized scores) that was also used for analyses of anogenital distance and of ovarian follicles because these data were not normally distributed. For analysis of sweet preference data, we summed the consumption of saccharin solution and that of water intake across the 5 days of testing and then calculated the ratio of saccharin to water intake to obtain a measure for the preference. These preference values were analyzed by two-way ANOVA with exposure as between-litter factor and sex as within-litter factor. This was followed by preplanned separate analyses of preference in males and females with one-way ANOVA and exposure as the between factor. We considered an error probability of *p* < 0.05 significant in all analyses.

## Results

### Developmental data.

Body weight of dams and body weight gain during gestation were not influenced by exposure to PBDE-99 or A1254 (Table 1). The number of implantations per litter, the number of pups per litter, and the percentage of male pups were not different from controls. On PND7, body weights were slightly reduced in male and female pups exposed to A1254 compared with controls (*p* < 0.05). No significant differences between A1254 and controls were found at other time points. The lower dose of PBDE-99 induced a slight increase in body weight in female pups at birth (*p* < 0.05) but not at later time points. Compared with A1254, both doses of PBDE-99 increased body weights in male pups in the first 2 weeks after birth (*p* < 0.05). In female pups, this was seen only at the lower dose of PBDE-99 and not in the second week of post-natal development. When litter size was included as a covariate in the analysis, the general pattern of effects did not change much. All significant effects remained significant, but a few other differences between A1254 and the PBDE-treated groups also became significant (*p* < 0.05) in both sexes (Table 1).

### Organ weights.

**Figure 1:**
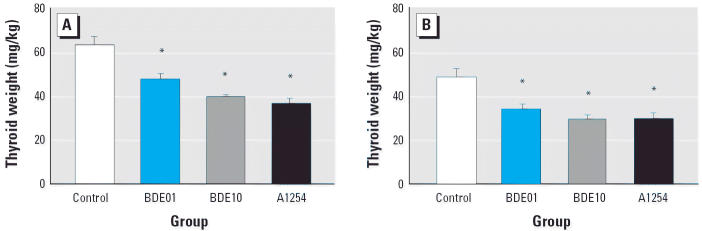
Thyroid weights in male (*A*) and female (*B*) adult offspring after exposure to low and high doses of PBDE-99 or A1254. Data shown are mean ± SE (*n* = 8/group). *p < 0.05 compared with control.

Organ weights are given in Table 2. At weaning, there were no significant differences between PBDE-exposed and control rats in weights of brain, thymus, testis, ventral prostate, and uterus. There was a tendency for decreased pituitary weights in male offspring at the higher dose level compared with controls (*p* < 0.1), whereas the difference between the higher dose and A1254 males was significant (*p* < 0.05). In contrast, females of the lower dose group exhibited significantly increased pituitary weights compared with controls (*p* < 0.05). A1254 exposure caused a significant decrease in thymus weight in male pups compared with controls (*p* < 0.05). The most remarkable effect seen in adult rats was the reduction in thyroid weights in both sexes after PBDE and A1254 exposure (Figure 1). This effect was significant even at the lower dose (*p* < 0.05) and more pronounced at the higher one. Mean ovary weights (± SE) were 408 ± 13, 393 ± 22, 388 ± 17, and 344 ± 16 mg/kg body weight in controls, BDE01, BDE10, and A1254 rats, respectively. The decrease approached significance in A1254 rats compared with controls (*p* < 0.1). There were no treatment-related effects on weights of other organs or body weights in adult rats.

### Anogenital distance and puberty onset.

Results for anogenital distance and puberty onset are shown in Table 3. Exposure to A1254 delayed puberty onset in both sexes (*p* < 0.05), whereas the higher dose of PBDE-99 caused a delay only in female offspring (*p* < 0.05). In contrast, the lower dose resulted in a slight acceleration of puberty onset in male rats compared with controls (*p* < 0.1). Significant differences between both PBDE groups and A1254 rats (*p* < 0.05) were found only in male rats, with puberty being later in the A1254 group.

At weaning, anogenital distance was decreased by A1254 exposure in both sexes compared with controls (females *p* < 0.05, males *p* < 0.1). In males, anogenital distance decreased with increasing dose of PBDE and approached statistical significance (*p* < 0.1) in the BDE10 group. These effects on anogenital distance were persistent in adult males at PND160 (Table 3). Anogenital distances of weanling female rats from both PBDE groups were significantly higher than those of the A1254 female pups (*p* < 0.05) but did not differ from controls.

### Ovarian follicles.

A statistically significant reduction in the primordial/primary follicle pool was observed in the BDE01 group (*p* < 0.05), and a dose-related decrease in the number of secondary follicles was seen in females exposed to PBDE-99 that was significant in the BDE10 group (*p* < 0.05). Exposure to A1254 resulted in a statistically significant increase in the number of tertiary follicles compared with controls (*p* < 0.05) (Table 4).

### Sex steroid concentrations.

**Figure 2:**
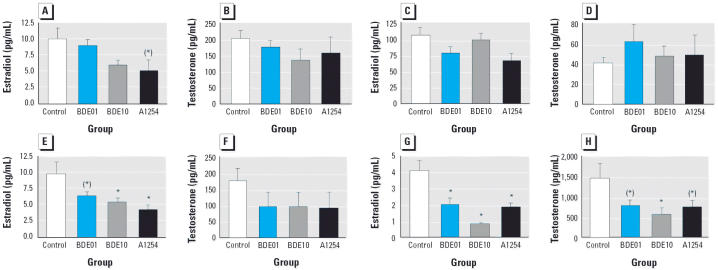
Serum concentrations (mean ± SE) of estradiol (*A*, *C*, *E*, *G*) and testosterone (*B*, *D*, *F*, *H*) in dams on PND21 (*A, B*), female offspring on PND21 (*C, D*), male offspring on PND21 (*E, F*), and male offspring on PND160 (*G, H*). *n* = 8/group. *p < 0.05 compared with control. (*)*p* < 0.1 compared with control.

Circulating concentrations of estradiol and testosterone are shown in Figure 2. Exposure-related differences in serum estradiol could be detected in dams on PND21 [*F*(3,25) = 3.33, *p* < 0.05]. Estradiol concentrations were decreased by about 37% and 53% in the BDE10 dams and A1254 dams, respectively. Post hoc tests were not significant, but the decrease in A1254 rats approached significance (*p* < 0.1; Figure 2A). Circulating testosterone was reduced in BDE10 dams and A1254 dams by 33% and 22%, respectively, but these differences did not reach statistical significance (Figure 2B). Differences in serum estradiol approached significance in female offspring on PND21 [*F*(3,26) = 2.36, *p* < 0.1], but post hoc tests failed to show any statistical differences (Figure 2C). Testosterone concentrations in female pups were not altered by exposure (*p* > 0.1; Figure 2D). In weanling males, circulating estradiol was reduced in all treatment groups [*F*(3,28) = 4.36, *p* < 0.05]. This reduction was more pronounced in the group exposed to the higher dose, being approximately 34% and 44% in BDE01 and BDE10 males, respectively. The reductions in A1254 males measured 57% compared with controls (Figure 2E). According to post hoc tests, the difference to controls approached significance in BDE01 males (*p* < 0.1) and was significant in BDE10 and A1254 males (*p* < 0.05). Circulating testosterone was reduced by about 50% of control values in all treated groups, but due to high variability these differences failed to reach significance (Figure 2F). Effects on circulating sex steroids were even more pronounced in adult males (Figure 2G, H). There were reductions in serum concentrations of estradiol [*F*(3,28) = 12.39, *p* < 0.0001] and testosterone [*F*(3,28) = 3.00, *p* < 0.05]. The reductions in estradiol concentrations were more pronounced at the higher dose, being 52% and 79% in BDE01 and BDE10 groups, respectively. A1254 males exhibited a reduction of 54%. Post hoc tests revealed significant differences for all treated groups compared with controls (*p* < 0.05). Testosterone was decreased by 45% and 60% in BDE01 and BDE10 males, respectively, and by about 50% in male rats exposed to A1254. Differences were significant between BDE10 males and controls according to post hoc tests (*p* < 0.05) and approached significance in BDE01 and A1254 males (*p* < 0.1).

### Sweet preference.

**Figure 3:**
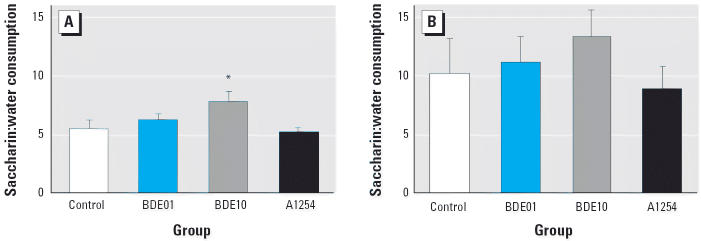
Saccharin-to-water consumption ratio (mean ± SE) in adult male offspring (*A*; *n* = 9–11/group) and adult female offspring (*B*; *n* = 10–12/group). *p < 0.05 compared with control.

Exposure to PBDE-99 caused significant increases in the ratio of saccharin-to-water consumption in male rats [Figure 3A; *F*(3,36) = 2.87; *p* < 0.05]. Values were increased by 14% and 45% in BDE01 and BDE10 groups, respectively, with a significant difference between BDE10 rats and controls according to post hoc tests (*p* < 0.05). Males exposed to A1254 did not differ from controls, but the deviation from BDE10 males was significant (*p* < 0.05). In general, the ratio of saccharin to water consumption was about 2-fold higher in female (Figure 3B) than in male rats (Figure 3A), showing that this behavior is indeed sexually dimorphic. PBDE-treated females exhibited some signs of supernormality, with increases of about 8% in the BDE01 group and 30% in the BDE10 group, but these differences were not significant.

### Analysis of PBDE-99 in tissues.

Results of PBDE-99 tissue levels are shown in Table 5. From the day after the last treatment (GD19) to weaning (PND21), the concentrations decreased in the brain and adipose of dams. On PND21, PBDE-99 concentrations in the brain reached control levels in BDE01 dams, whereas there was a reduction to approximately 25% of initial values in brains of BDE10 dams. Decreases of approximately 48% and 10% were observed in adipose tissue of BDE01 and BDE10 dams, respectively. Compared with those of dams, brain levels of PBDE-99 were lower by factors of 7–9 in offspring on GD19. Similar to those of dams, brain concentrations declined to control values in low-dosed male and female offspring by PND21. In the BDE10 group, brain concentrations in male and female offspring were lower than those of dams by factors of 3–7. In adipose tissue, the concentrations were lower by factors of 6–17 and 13–38 in BDE01 and BDE10 offspring, respectively. Internal PBDE concentrations in males were slightly higher than in females on PND21, but the differences are still within analytical statistical variation. On PND160, brain concentrations were indistinguishable from controls in both PBDE-treated groups, whereas there was still a detectable elevation in adipose tissue of BDE10 males.

## Discussion

Gestational exposure to PBDE-99 did not affect reproductive success in dams or development of body weights in offspring at the doses tested. The weights of nonreproductive and reproductive organs were largely unchanged; however, we found increased pituitary weights at the low dose in female offspring and decreased weights at the high dose in male offspring. This may indicate a biphasic response to PBDE exposure, in particular, because we found small nonsignificant increases in low-dose males, as well. A similar biphasic response has been reported for anterior pituitary weights in male rats after exposure to the commercial PBDE mixture DE-71 using a pubertal protocol (Stoker et al. 2004). In our rats, these changes were transient, because they were not observed in adulthood in the male offspring.

A marked effect was the decrease in thyroid weights in adult offspring, which was more pronounced in the high-dose group. However, PBDE effects on the thyroid were not the main focus of our study. There are several reports on decreased circulating concentrations of thyroid hormones after developmental exposure to PBDE mixtures and single congeners in mice and rats (Kuriyama et al. 2005; Skarman et al. 2004; Zhou et al. 2002). The reasons for decreased thyroid hormones are not entirely clear because the proposed mechanisms—induction of thyroid-metabolizing uridine diphosphate-glucuronosyltransferase (UDP-GT) and competitive binding to serum transport proteins—are not sufficient to explain hormone reductions in certain animal models after exposure to PCBs (Kato et al. 2004). Reduced thyroid hormone concentrations recover within 2–3 weeks after termination of exposure of rats after weaning (Stoker et al. 2004; Zhou et al. 2002), whereas in the present study we found the reduced thyroid weights in adult rats long after the end of the developmental treatment when internal concentrations of PBDE-99 had declined to nearly control values.

The delay of puberty onset we observed in female rats at the higher dose of PBDE-99 is in accordance with results of pubertal exposure to the technical PBDE mixture DE-71 (Stoker et al. 2004). However, in males we detected a small acceleration at the lower dose of PBDE-99, in contrast to the dose-dependent delay reported for DE-71. This discrepancy may be related to the different substances used for exposure or to the different exposure periods. Although PBDE-99 is the major constituent of DE-71 (48%), only a slightly smaller proportion of this mixture is composed of tetrabrominated PBDE-47 (37%) and pentabrominated PBDE-100 (8%) (Sjödin et al. 2003). These latter congeners, like DE-71, are reported to inhibit dihydrotestosterone-induced transcriptional activation of the human androgen receptor (AR) *in vitro* in the micromolar range. This was not observed for PBDE-99, although it exhibits competitive binding to the AR (Stoker et al. 2005). Thus, the different antiandrogenic activities of DE-71 and PBDE-99 may underlie the divergent effects observed for puberty onset in males. On the other hand, the influence of different exposure periods (prenatal versus pubertal) cannot be excluded. The mechanism behind the accelerated puberty onset in males exposed to PBDE-99 remains to be determined. Because both testosterone and estradiol levels were reduced in male offspring, the observed effect may be due to an imbalance in the relation of androgen to estrogen levels during prenatal and early postnatal development. Alternatively, PBDE-99 may interact with epidermal growth factors that have been shown to result in accelerated preputial separation (Henck et al. 2001) via stimulation of the release of gonadotropin releasing hormone by prostaglandin-E2 (Ojeda and Ma 1998). The reductions in circulating concentrations of sex steroids in the weanling and adult male offspring resemble similar decreases found after exposure to a reconstituted PCB mixture (Hany et al. 1999; Kaya et al. 2002). Although several actions on enzymes involved in steroid synthesis and metabolism have been described for PCBs, such data are lacking for PBDEs. In the present study, decreased steroid concentrations became more pronounced in adult males, thus demonstrating a long-lasting effect.

Interplay between the hypothalamo–pituitary–thyroid axis and the ovary has been demonstrated as disturbed folliculogenesis has been reported in mice and rats treated postnatally with 6-propyl-2-thiouracil, an agent used to treat hyperthyroidism (Chan and Ng 1995; Dijkstra et al. 1996). In addition, intraperitoneal administration of 2.5 mg Aroclor 1016/kg/day on GD7–13 (Baldridge et al. 2003) led to a reduction in preantral and antral follicles, with the reduction not occurring in the preantral group when thyroxine was simultaneously administered with Aroclor 1016. Similar to these findings, we observed a reduction in the primordial/primary follicles in the BDE01 group and a decrease in secondary follicles in the BDE10 group. A functional consequence of a decrease in the resting follicle pool could be a shorter reproductive life span because the size of the resting follicle pool is believed to be a major determinant of ovarian senescence. We observed an increased number of tertiary follicles in the A1254 group, indicating altered maturation or prolongation of this phase by postponement of atresia. Thus, the observed changes in follicle numbers may be related to thyroid effects because thyroid weights were decreased in exposed rats. On the other hand, the alterations in folliculogenesis and prepubertal steroidogenesis may indicate disruption along the hypothalamo–pituitary–ovarian axis after exposure to either PBDE-99 or A1254. Follicle-stimulating hormone (FSH) is responsible for follicular growth and differentiation of granulose cells that surround oocytes during the entire follicular development. Therefore, changes in gonadotropin levels or a direct effect of PBDE on granulosa cells, leading to alterations in FSH receptors on these cells, could affect follicular maturation. Influences on gonadotropin concentrations were described after exposure to PCBs in female (Lyche et al. 2004) and male mammals (Desaulniers et al. 1999). Additional data are needed to clarify if effects on the production of FSH or alterations in FSH receptors are involved in disturbances of folliculogenesis by PBDE-99.

The increased sweet preference seen after exposure to PBDE-99 in adult male offspring is similar to effects seen after treatment with a PCB mixture reconstituted according to the congener pattern found in human milk (Hany et al. 1999; Kaya et al. 2002). This behavior is sexually dimorphic, with females demonstrating a higher preference for sweet, such that an elevation in the intake of the sweetened water by the males indicates behavioral feminization. For PCBs, increased sweet preference was related to influences on sexual differentiation of the brain, demonstrated by decreased activity of hypothalamic aromatase in newborn male pups (Hany et al. 1999). The peak of aromatase activity is around birth, and the resultant increase in the local conversion of testosterone to estradiol is one of the key processes in the male-like differentiation of the rodent brain and subsequent sexually dimorphic behavior (e.g., Hutchison et al. 1999; Negri-Cesi et al. 2004). At present, there are no data on aromatase activity in developing rats exposed to PBDE-99, but preliminary results of a study following the same protocol as in the present study indicate alterations in steroid receptor mRNA levels in brain regions related to sexual functions in rats exposed to PBDE-99 (Lichtensteiger et al. 2003).

The PBDE-99 used in the present study has been analyzed for impurities, and all results for brominated dioxins, furans, and coplanar polybrominated biphenyls were below the detection limits. However, in the worst case this could mean that the PBDE-99 used might contain brominated dibenzofurans and dioxins at a level of about 0.50 μg TEQ/g PBDE-99. This would have resulted in a total TEQ exposure of 4.5 and 45 ng/kg body weight in the BDE01 and BDE10 groups, respectively. However, a much higher TEQ value of 39.42 μg/g was reported for a low-contaminated lot of A1254 (Kodavanti et al. 2001), and the A1254 doses used in our study were 3- and 30-fold higher than the respective doses of PBDE-99. Moreover, some of the end points in this study, such as pituitary weight and sweet preference in male offspring and primary and secondary follicles in female offspring, were affected by PBDE-99 but not by A1254. The lack of ability of A1254 to alter sweet preference has been previously detected in our laboratory (Hany et al. 1999). In that study, A1254 was compared with a PCB mixture that was reconstituted according to the congener pattern found in human breast milk and included coplanar and mono-*ortho*-chlorinated PCBs. Thus, differences in TEQ content are unlikely to account for the results. Moreover, developmental exposure to 2,3,7,8-tetrachlorodibenzo-*p*-dioxin (TCDD) or coplanar PCBs did not affect sweet preference in male rats but caused masculinization of this behavior in female rats (Amin et al. 2000). This suggests that the influence of PBDE-99 on sweet preference in the present study cannot be ascribed to aryl hydrocarbon (Ah) receptor activity of contaminants. There are other effects of PBDEs for which mediation by Ah receptor activity is not likely. Only PBDE-99, and not A1254, was reported to cause apoptotic cell death in astrocytoma cells (Madia et al. 2004), and the PBDE mixture DE-71 caused disturbance of protein kinase C activation and calcium homeostasis in neuronal cell preparations, measures that are not influenced by coplanar PCBs (Kodavanti et al. 2005).

From the tissue concentration data, it appears that PBDE-99 is more rapidly removed than are PCBs. Values in the brain of offspring were highest at GD19. This is in contrast to previously reported results with the reconstituted PCB mixture (Kaya et al. 2002; Lilienthal et al. 2000), where there was an increase in brain concentrations in offspring until weaning. Also, the decline of PBDE-99 concentrations in adipose tissue of dams during lactation and in male offspring from weaning to adulthood appears to be more pronounced. Further investigations should clarify if this holds true only for PBDE-99 or if PBDE congeners are generally less stable in the mammalian body than their respective PCB congeners. The internal exposure values found in adipose tissue of dams in this study are higher by factors of about 8–15 in the low-exposure group than the 95th percentile of the total PBDE concentration found in human breast milk in the United States (Schecter et al. 2003). In Europe, these factors are approximately 10–100 times higher than in North America (Meironyte Guvenius et al. 2001; Meneses et al. 1999).

## Conclusions

The present results indicate an endocrine-modulating activity of PBDE-99. Effects on several end points, including anogenital distance, sex steroid concentrations, and sweet preference in male rats as well as ovarian follicle number in female rats, were detected in adulthood long after the termination of exposure demonstrating the persistence of effects after developmental treatment. Some of the alterations induced by PBDE-99 are not likely to be due to TEQ impurities because A1254 and PBDE-99 exposure resulted in different patterns of effects.

## Figures and Tables

**Table 1:** Reproductive data of rats exposed to PBDE or A1254 (mean ± SE).

	Age	Control	BDE01	BDE10	A1254
Dam body weight (g)	GD8	203.7 ± 5.3	198.8 ± 5.3	203.9 ± 4.8	200.1 ± 5.8
	GD10	214.7 ± 5.1	207.4 ± 4.9	212.5 ± 4.5	208.4 ± 5.4
	GD20	279.8 ± 7.9	276.9 ± 5.2	282.8 ± 7.1	275.5 ± 6.8
Implantations		12.2 ± 0.5	12.1 ± 0.5	12.8 ± 0.4	11.9 ± 0.4
No. of litters		25	26	26	22
Pups per litter		10.4 ± 0.4	10.8 ± 0.4	11.8 ± 0.4	10.9 ± 0.5
Percent male		48.1	50.9	52.5	49.2
Pup body weight (g)	PND0	5.9 ± 0.1	6.0 ± 0.1**	5.9 ± 0.1#	5.7 ± 0.1
Male	PND7	14.0 ± 0.3	14.4 ± 0.2**	13.8 ± 0.3**	12.4 ± 0.3*
	PND14	26.4 ± 0.5	27.0 ± 0.3**	26.8 ± 0.5**	25.1 ± 0.5
	PND21	41.2 ± 1.5	42.1 ± 1.3#	41.9 ± 1.2#	37.7 ± 1.1
	PND0	5.5 ± 0.1	5.7 ± 0.1*^,^**	5.5 ± 0.1	5.3 ± 0.1
Female	PND7	14.2 ± 0.7	13.7 ± 0.2**	13.2 ± 0.3#	11.9 ± 0.3*
	PND14	25.2 ± 0.6	26.0 ± 0.3#	25.6 ± 0.4	24.5 ± 0.5
	PND21	39.0 ± 1.5	40.0 ± 1.2	39.7 ± 1.1#	36.3 ± 1.2

* *p* < 0.05 compared with control.

** *p* < 0.05 compared with A1254.

# *p* > 0.05 compared with A1254 after analysis of covariance with litter size as the covariate.

**Table 2:** Relative organ weights (mean ± SE) in dams and weanling offspring.

	Organ	Control	BDE01	BDE10	A1254
Dams					
GD19	Brain	6.5 ± 0.2	6.6 ± 0.1	6.3 ± 0.1	6.8 ± 0.1
	Pituitary	38.8 ± 6.0	34.4 ± 4.6	33.4 ± 4.4	33.7 ± 6.0
PND21	Brain	6.9 ± 0.2	7.1 ± 0.2	7.1 ± 0.2	6.8 ± 0.3
	Pituitary	36.4 ± 5.1	38.3 ± 6.9	41.2 ± 2.4	45.0 ± 2.9
	Liver	51.9 ± 1.5	53.7 ± 1.5	52.1 ± 1.3	56.6 ± 1.6
Offspring, PND21					
Female	Brain	35.8 ± 0.4	38.2 ± 1.4	35.2 ± 0.4	39.8 ± 4.8
	Pituitary	73.4 ± 5.6	99.7 ± 9.5*	69.5 ± 7.9	84.0 ± 4.6
	Thymus	3.76 ± 0.15	3.77 ± 0.10	3.80 ± 0.11	3.39 ± 0.17
Male					
	Brain	33.7 ± 2.9	34.9 ± 1.0	32.7 ± 1.0	35.5 ± 0.5
	Pituitary	61.3 ± 11.4	72.7 ± 6.4	41.8 ± 5.4 (*)^,^**	75.8 ± 6.8
	Thymus	3.37 ± 0.19	3.79 ± 0.07**	3.43 ± 0.14**	2.80 ± 0.20*

Brain, liver, and thymus weights are given in g/kg body weight; pituitary, uterus, and ovary weights are given in mg/kg body weight (GD19, *n* = 3/group; PND21, *n* = 6–8/group).

* *p* < 0.05 compared with control.

* *p*) < 0.1 compared with control.

** *p* < 0.05 compared with A1254.

**Table 3:** Development of reproductive organs given as median (first quartile, third quartile); *n* = 8/group.

	Age	Control	BDE01	BDE10	A1254
Male offspring					
Puberty onset (PND)		44.7 (44.0, 44.8)	44.3(*)^,^# (43.0, 44.4)	44.3# (43.3, 45.0)	45.3* (45.0, 47.5)
Anogenital distance	PND21	3.57 (3.23, 3.79)	3.40 (3.21, 3.61)	3.26(*) (3.08, 3.41)	3.34(*) (3.04, 3.40)
	PND160	6.95 (6.79, 7.12)	6.66 (6.53, 6.97)	6.69(*) (6.37, 6.99)	6.48* (6.18, 6.89)
Female offspring					
Puberty onset (PND)		33.7 (33.6, 33.8)	34.2 (33.8, 34.3)	34.0* (33.9, 34.8)	34.4* (34.2, 34.7)
Anogenital distance	PND21	2.03 (1.66, 2.08)	1.95# (1.69, 2.16)	1.83# (1.71, 2.03)	1.50* (1.41, 1.77)

Puberty onset is given for PND of preputial separation in males or vaginal opening in females (*n* = 10 litters/group). Anogenital distance was standardized to the cubic root of body weight and is given as 10 × mm ÷ g^1/3^.

* *p* < 0.05 compared with control,

(*) *p* < 0.1 compared with control, and

# *p* < 0.05 compared with A1254, by Van der Waerden test.

**Table 4:** Median ovarian follicle numbers in 220-day-old female offspring given as median (first quartile, third quartile); *n* = 8/group.

	Primordial/primary	Secondary	Tertiary	Atretic
Control	48 (46.5, 59.0)	12 (8.0, 13.5)	6 (5.0, 9.0)	2 (1.5, 2.5)
BDE01	34 (28.5, 45.0)*	9 (6.5, 14.5)	10 (7.5, 11.5)	2 (0.0, 4.0)
BDE10	47 (35.5, 64.0)	6 (5.5, 8.0)*	8 (7.0, 9.0)	2 (1.5, 3.5)
A1254	42 (34.0, 52.0)	11 (4.5, 13.5)	10 (8.5, 11.5)*	2 (1.0, 3.0)

* *p* < 0.05 compared with control, by Van der Waerden test.

**Table 5:** Tissue concentrations (mean ± SE, μg/g wet weight) of PBDE-99 (*n* = 3/group).

	Age	Control	BDE01	BDE10	A1254
Dams					
Brain	GD19	0.010 ± 0.004	0.372 ± 0.034	4.090 ± 0.319	0.005 ± 0.003
	PND21	0.005 ± 0.001	0.018 ± 0.007	1.148 ± 0.615	0.016 ± 0.003
Adipose	GD19	0.015 ± 0.005	5.103 ± 1.754	88.200 ± 23.477	0.014 ± 0.003
	PND21	0.010 ± 0.008	2.672 ± 1.430	78.200 ± 32.456	0.010 ± 0.005
Offspring					
Brain	GD19	0.013 ± 0.004	0.053 ± 0.008	0.465 ± 0.140	0.010 ± 0.002
Female offspring					
Brain	PND21	0.024 ± 0.017	0.021 ± 0.003	0.175 ± 0.062	0.021 ± 0.012
Adipose	PND21	0.031 ± 0.020	0.159 ± 0.031	2.052 ± 0.949	0.003 ± 0.001
Male offspring					
Brain	PND21	0.036 ± 0.022	0.036 ± 0.008	0.376 ± 0.139	0.048 ± 0.016
	PND160	0.006 ± 0.003	0.003 ± 0.001	0.008 ± 0.006	0.004 ± 0.002
Adipose	PND21	0.019 ± 0.001	0.429 ± 0.207	6.020 ± 1.859	0.009 ± 0.004
	PND160	0.003 ± 0.001	0.026 ± 0.002	0.140 ± 0.003	0.004 ± 0.001
